# Struggling with extensive informed consent procedures for cancer trials—is there even a benefit for the patients?

**DOI:** 10.1007/s00520-022-07063-w

**Published:** 2022-04-29

**Authors:** Marie-Kristin Tilch, Melanie Schranz, Alice Moringlane, Matthias Theobald, Georg Hess

**Affiliations:** 1 Department of Hematology, Oncology and Pneumology & University Cancer Center, University Medical Center of the Johannes Gutenberg University, Langenbeckstr. 1, 55131 Mainz, Germany; 2 Institute of Medical Biostatistics, Epidemiology and Informatics, University Medical Center of the Johannes Gutenberg University, Mainz, Germany; 3 Department of Anesthesia, Klinikum Kassel, Kassel, Germany

**Keywords:** Cancer, Informed consent, Comprehension, Patient education

## Abstract

**Purpose:**

Informed consent procedures in clinical trials often differ in length and complexity to those in clinical routine care. Little is known about the benefit of extensive procedures as intended in clinical trials compared to procedures in routine cancer treatment.

**Methods:**

In two different clinical studies performed at a comprehensive cancer center, we compared patients’ comprehension and satisfaction of current informed consent procedures in routine clinical care with the level of comprehension and satisfaction of patients treated within clinical trials. Patients with a new cancer diagnosis and recent informed consent received a questionnaire about satisfaction, comprehension, time management, and physician–patient relationship of the informed consent process. Patients in cohort 1 consented to cancer treatment within a clinical trial and were additionally interviewed in a structured way; patients in cohort 2 consented to “standard” chemotherapy and received a follow-up questionnaire after 6 months.

**Results:**

In cohort 1, 82 patients completed the questionnaire and had an additional structured interview. They were treated in 41 different trials, receiving up to 40 pages of educational material. In cohort 2, 89 patients completed the first and 52 completed the follow-up questionnaire after receiving a standard informed consent form of 6 pages. Subjective understanding and satisfaction with the information provided was equally very high. However, deficits in objective understanding were observed in both cohorts.

**Conclusion:**

Extensive informed consent procedures for clinical cancer trials have not been associated with a higher level of satisfaction or measurable objective understanding; therefore, the benefit seems to be limited.

## Background

With increasing progress in cancer treatment, informed consent procedures including written educational material have become more and more complex within the past decades. Legal requirements impose full disclosure of all relevant information concerning different therapeutic options, effects, risks, and potential side effects. Procedures can be quite time-consuming and challenging, since sociodemographic factors (sex, age, cultural background) as well as individual cognitive capacities and physical conditions have to be considered [1]. These extensive explanations aim to guarantee full comprehension and hereby respect patient autonomy [2], but in fact, this often exceeds individual capacities and expectations, resulting in “over-informedness.” However, the use of written informed consent forms is a standard procedure, although their benefit has often been questioned due to problems with length, format, challenging diction, and readability, especially for lay persons and patients with disabilities [3–5].

Especially in clinical trials, as the extended length of the educational material can keep patients from reading the material completely, comprehension may suffer [6]. Misunderstanding of key aspects of patient information has been observed in both patients in routine cancer care and in trial participants, e.g., misconception of cancer-related terminology or of potential risks of the trial or of its design [7–9]. This is critical, since inadequate communication in cancer therapy can impair the patient-doctor relationship, compliance, and engagement during therapy and additionally impacts satisfaction, quality of life, and health outcomes [3–5, 10].

Supposing that current informed consent procedures in routine cancer treatment are already satisfactory, it can be assumed that the benefit of more detailed and complex informed consent procedures for patients treated in clinical cancer trials is rather limited and comprehension is not further improved. This question was explored in a prospective study at a Comprehensive Cancer Center (CCC), which investigated patients’ satisfaction and understanding regarding informed consent procedures and materials in the context of clinical trials (survey 1). The results of the study prompted the need to further investigate patients’ understanding and satisfaction in the context of standard cancer care (survey 2). To compare potential differences in comprehension and satisfaction after informed consent in the context of a standard cancer care with a cohort treated within a clinical cancer trial, we prospectively investigated these issues in cancer patients treated at the same CCC. In this subsequent study, we analyzed patients’ perceptions of the informed consent process in routine cancer care in terms of comprehension and satisfaction, showing that information needs are generally high and some needs are not being met, though patients generally expressed overall satisfaction and considered their subjective comprehension to be good [11]. We could demonstrate that increasing age and a lower education level have a clear impact on patients’ comprehension, which is in line with findings from prior studies [12, 13].

## Methods

Survey 1 was conducted between 2013 and 2015 at the CCC Mainz, Germany. For this prospective study, 97 patients with a new diagnosis of hematological or solid malignancy with need for systemic therapy and treatment option within a clinical trial were screened. Eligibility criteria included the following: age ≥ 18 years, written informed consent, informed consent talk within the past 2 days but no longer than 3 weeks ago, and sufficient comprehension of the German language. Patients gave written informed consent on trial-specific consent forms. At study inclusion, sociodemographic factors such as profession and marital status were collected. Patients were asked to complete a questionnaire containing 53 items subdivided into the following dimensions: quality and extent of the informed consent process, reading time, associated decision time, and patient-doctor relationship. The questionnaire was developed, piloted, and finalized based on already published and validated questionnaires like the EORTC QLQ-INFO25 questionnaire [14] and the Qualiskope-A questionnaire [15] (“Analysis of patients’ satisfaction with informative material in the field of oncology,” A. Moringlane, 2017, unpublished doctoral thesis). In total, 43 questions were designed according to a 10-point Likert scale, 6 questions were designed according to an ordinal scale, 2 questions were dichotomous, and 2 questions were to be answered as a free text (e.g., “name your disease,” “which organ is mainly affected?”). After completing the questionnaire, a structured interview with 27 questions was performed by the same interviewer in all patients. It consisted of 9 questions designed according to a 10-point Likert scale, 2 questions designed according to an ordinal scale, 7 dichotomous questions, and 9 questions to be freely answered, some of them testing the knowledge directly (“what is a randomized trial?”; “what is the goal of the trial?”; “who is the sponsor of the trial?”). For easier evaluation, the questions in the questionnaire and in the interview were grouped into three response categories: “rather not agree” and “not agree at all” (1–3 points on the 10-point Likert scale), “partial agreement” (4–7 points on the 10-point Likert scale), and “rather agree” or “fully agree” (8–10 points on the 10-point Likert scale).

Survey 2 was subsequently conducted between September and December 2015 at the CCC Mainz, Germany [11]. For this prospective study, 100 patients with a new diagnosis of hematological or solid malignancy with need for systemic therapy without available clinical trial options were screened. Eligibility criteria and sociodemographic factors collected were the same as in survey 1. All patients gave written consent using the standardized consent form on cytostatic system therapy, which is used by the CCC, Mainz, Germany. The initial questionnaire was optimized, reducing it to 40 questions by removing redundant or irrelevant questions and changing the answer options to a 5-point Likert scale for a more user-friendly design and to avoid random answers because of too many gradations. The shortenings and different scaling had no influence on the evaluation of the most relevant questions. It was tested with 16 cancer patients before starting the trial at the same hospital. In total, 20 questions were designed according to a 5-point Likert scale, 8 questions were designed according to an ordinal scale, 6 questions were dichotomous, and 6 questions were to be answered as a free text. The interview performed in cohort 1 revealed no additional findings; therefore, it was eliminated in cohort 2. Instead, an additional follow-up questionnaire with 9 questions was included to be filled in after 6 months, to evaluate the informed consent process retrospectively. For a coherent evaluation in line with the three response categories from survey 1, we also regrouped our five response categories into three.

### Statistical analysis

Sociodemographic characteristics (e.g., age, sex, education) and medical characteristics (e.g., therapy goal, tumor type) were analyzed with descriptive statistics. Continuous variables are presented as median and interquartile range (IQR); categorical variables are provided as absolute numbers and corresponding percentages. For survey 2, patients who completed both questionnaires were compared to patients who had dropped out of the survey. This comparison was performed using the Mann–Whitney *U* test (for continuous variables) and the Exact Fisher test (for categorical variables). Interrelations between influencing variables and response patterns were investigated by means of analysis of variance. The mean value and the standard deviation were determined and specified, when needed. For all tests, we used a 0.05 level to define statistically relevant deviation from the respective null hypothesis. Data were analyzed using IBM SPSS Statistic Version 23.0 (IBM Corp., Armonk, NY, USA).

## Results

### Patients and treatment

For survey 1, 97 patients were screened, of which 82 patients were finally enrolled and completed the questionnaire, while 15 patients refused to complete the questionnaire after explaining the concept of the survey (reasons unknown). 41.5% (*n* = 34) of the patients suffered from solid malignancies (cancer of the gastrointestinal tract, pancreas, lung, biliary tract, ovaries, and breast) and 58.5% (*n* = 48) of the patients suffered from hematological malignancies (acute or chronic myeloid or lymphatic leukemia, myelodysplastic syndrome, multiple myeloma, aplastic anemia, and Hodgkin and non-Hodgkin lymphoma). They were treated within 41 different clinical trials (phase II–IV) with curative therapy goals. 22.5% of the patients had been treated within a clinical trial before. Informed consent material was between 14 and 40 pages.

For survey 2, 100 patients were screened, of which 89 patients were finally enrolled and completed the questionnaire. The 11 patients who were excluded either declined the participation, were too ill to participate, or did not meet the inclusion criteria. 56% (*n* = 50) of the patients suffered from solid malignancies (cancer of the gastrointestinal tract, pancreas, lung, biliary tract, brain, skin, soft tissue, bone) and 44% (*n* = 39) of the patients suffered from hematological malignancies (acute or chronic myeloid or lymphatic leukemia, and Hodgkin and non-Hodgkin lymphoma). 45% of the patients had a curative, 7% an adjuvant, and 45% a palliative therapy goal. In 3% of the cases, the treatment goal was not clearly defined at the time of study inclusion. The standardized informed consent form contained 6 pages. Additional baseline characteristics of both cohorts were similar regarding gender distribution, age, and school education and are displayed in Table 1.

**Table 1:** Baseline characteristics of both cohorts at study inclusion

	Cohort 1	Cohort 2
Number of patients	82 (100%)	89 (100%)
Median age (IQR)	58.3 (30–83)	58.6 (18–82)
Sex		
Male	54 (66%)	55 (62%)
Female	28 (34%)	34 (38%)
Years of school education		
Thirteen	29 (35%)	27 (30%)
Ten	18 (22%)	22 (25%)
Nine	35 (43%)	28 (32%)
Unknown	0 (0%)	12 (13%)
Therapy goal		
Curative	82 (100%)	39 (45%)
Adjuvant	0 (0%)	8 (7%)
Palliative	0 (0%)	39 (45%)
Unknown	0 (0%)	3 (3%)

### Questionnaire

#### Duration of the informed consent process

In both surveys, most patients had only one single conversation with their physician prior to therapy start (survey 1: 51%; survey 2: 53%). In survey 2, oral explanations usually took about 15–30 min (43%) or 30–60 min (22%) (unknown for survey 1), but in both cohorts patients reported high satisfaction with the time the doctor took to explain all relevant items (Table 2).

**Table 2:** Evaluation of understanding and satisfaction of the informed consent process at study initiation

	Cohort 1	Cohort 2	
	Absolute number of patients (percentage)	Absolute number of patients (percentage)	Absolute risk difference (95% CI)
Setting of the informed consent process			
How many conversations did you have with your doctor before signing the informed consent form?	*N* = 80	*N* = 89	
One	41 (50%)	47 (53%)	− 1.6% (− 16.3%; + 13.2%)
Two	30 (38%)	26 (29%)	+ 8.39% (− 5.8%; + 22.1%)
More than two	9 (11%)	16 (18%)	− 6.7% (− 17.4%; + 4.3%)
How much time did you need to read the informed consent material?	*N* = 82	*N* = 75	
Up to 15 min	33 (40%)	28 (38%)	+ 2.91% (− 12.2%; + 17.7%)
15–30 min	21 (26%)	31 (41%)	− 15.7% (− 29.7%; − 0.98%)
30–60 min	14 (17%)	4 (5%)	+ 11.75% (+ 1.7%; + 21.84)
More than 60 min	5 (6%)	4 (5%)	+ 0.76% (− 7.6%; + 8.8%)
I did not read it	6 (7%)	8 (11%)	− 3.35% (− 13.2%; + 5.95%)
No answer	3 (4%)	0 (0%)	+ 3.7% (− 1.8%; + 10.2%)
I read the informed consent material completely	*N* = 82	*N* = 89	
Rather or fully agree	61 (74%)	53 (60%)	+ 14.8% (+ 0.7%; + 28.1%)
Partially agree	9 (11%)	20 (22%)	− 11.5% (− 22.5%; − 0.14%)
Rather not agree or do not agree at all	12 (15%)	16 (18%)	− 3.34% (− 14.4%; + 8.0%)
I just wanted to sign the informed consent form	*N* = 81	*N* = 87	
Rather or fully agree	9 (11%)	25 (29%)	− 17.6% (− 29.1%; − 5.5%)
Partially agree	9 (11%)	17 (19%)	− 8.4% (− 19.3%; + 2.7%)
Rather not agree or do not agree at all	63 (78%)	45 (52%)	+ 26.1% (+ 11.6%; + 38.94)
I received a lot of additional information through (study) nurses	*N* = 72	*N* = 87	
Rather or fully agree	33 (46%)	14 (17%)	+ 29.7% (+ 15.5%; + 42.8%)
Partially	28 (39%)	21 (24%)	+ 14.6% (+ 0.3%; + 28.7%)
Rather not agree or do not agree at all	11 (15%)	52 (60%)	− 44.5% (− 56.2%; − 30.0%)
Comprehension			
The consent form was easy to understand	*N* = 80	*N* = 84	
Rather or fully agree	56 (70%)	60 (72%)	− 1.4% (− 15.2%; + 12.3%)
Partially	18 (22%)	18 (21%)	+ 1.1% (− 11.5%; + 13.8%)
Rather not agree or do not agree at all	6 (8%)	6 (7%)	+ 0.4% (− 8.2%; + 9.2%)
I had to ask a lot of questions to understand the informed consent form	*N* = 81	*N* = 82	
Rather or fully agree	9 (11%)	8 (9%)	+ 1.4% (− 8.4%; + 11.2%)
Partially agree	19 (24%)	19 (23%)	+ 0.3% (− 12.6%; + 13.2%)
rather not agree or do not agree at all	53 (65%)	55 (68%)	− 1.6% (− 15.9%; + 12.7%)
There were many incomprehensible words in the text	*N* = 75	*N* = 82	
Rather or fully agree	12 (16%)	11 (13%)	+ 2.6% (− 8.6%; + 14.1%)
Partially agree	21 (28%)	28 (34%)	− 6.2% (− 20.1%; + 8.3%)
Rather not agree or do not agree at all	42 (56%)	43 (53%)	+ 3.6% (− 11.8%; + 18.7%)
The consent form was well structured	*N* = 73	*N* = 82	
Rather or fully agree	53 (73%)	62 (76%)	− 3.0% (− 16.8%; + 10.6%)
Partially agree	19 (26%)	17 (21%)	+ 5.3% (− 7.9%; + 18.6%)
Rather not agree or do not agree at all	1 (1%)	3 (3%)	− 2.3% (− 8.9%; + 4.2%)
I know the purpose of the trial	*N* = 82	Not applicable	
Rather or fully agree	73 (89%)		
Partially agree	7 (9%)		
Rather not agree or do not agree at all	2 (2%)		
The therapy goal was clearly discussed with me	Not applicable	*N* = 88	
Rather or fully agree		66 (75%)	
Partially agree		16 (18%)	
Rather not agree or do not agree at all		6 (7%)	
Satisfaction			
I felt reassured after the informed consent	*N* = 77	*N* = 88	
Rather or fully agree	15 (12%)	43 (48.5%)	− 29.4% (− 42.0%; − 15.0%
Partially	36 (28%)	30 (34%)	+ 12.7% (− 2.3%; + 26.9%)
Rather not agree or do not agree at all	26 (20%)	15 (17.5%)	+ 16.7% (+ 3.5%; + 29.6%)
Chances and risks were well presented	*N* = 74	*N* = 89	
Rather or fully agree	53 (72%)	67 (75%)	− 3.7% (− 17.3%; + 9.7%)
Partially	19 (25%)	16 (18%)	+ 7.7% (− 4.9%; + 20.5%)
rather not agree or do not agree at all	2 (3%)	6 (7%)	− 4.0%; (− 11.5%; + 3.51%)
It was important to me to be informed about all side effects	*N* = 76	*N* = 88	
Rather or fully agree	53 (70%)	74 (84%)	− 14.4% (− 27.0%; − 1.5%)
Partially agree	12 (16%)	11 (13%)	+ 3.3% (− 7.4%; + 14.5%)
Rather not agree or do not agree at all	11 (14%)	3 (3%)	+ 11.1% (+ 2.3%; + 20.9%)
The doctor took enough time to explain everything	*N* = 82	*N* = 89	
Rather or fully agree	80 (98%)	76 (85%)	+ 12.2% (+ 3.7%; + 21.1%)
Partially	1 (1%)	9 (10%)	− 8.9% (− 17.0%; − 1.8%)
Rather not agree or do not agree at all	1 (1%)	4 (5%)	− 3.3% (− 9.9%; + 2.8%)
I had enough time to overthink my agreement to therapy	*N* = 82	*N* = 84	
Rather or fully agree	65 (79%)	65 (77%)	+ 1.9% (− 10.7%; + 14.3%)
Partially agree	8 (10%)	10 (12%)	− 2.15% (− 76.6; − 54.6%)
Rather not agree or do not agree at all	9 (11%)	9 (11%)	+ 0.26% (− 12.0%; + 7.7%)
Retrospectively, the informed consent displayed things decently		*N* = 43	
Fully agree		22 (52%)	
Rather agree		16 (37%)	
Partially		3 (7%)	
Rather not agree		1 (2%)	
Do not agree at all		1 (2%)	
Retrospectively, I am satisfied with the information I received		*N* = 43	
Fully agree		20 (47%)	
Rather agree		19 (44%)	
Partially		3 (7%)	
Rather not agree		1 (2%)	
Do not agree at all		0 (0%)	
I would have liked more information after all		*N* = 43	
Fully agree		3 (7%)	
Rather agree		4 (9%)	
Partially		11 (26%)	
Rather not agree		15 (35%)	
Do not agree at all		10 (23%)	
The therapy proceeded in the same way as the informed consent talk anticipated		*N* = 43	
Fully agree		19 (44%)	
Rather agree		17 (40%)	
Partially		6 (14%)	
Rather not agree		1 (2%)	
Do not agree at all		0 (0%)	

Regarding the time needed to read the information material, most patients in survey 1 took less than 15 min (40%) or between 15 and 30 min (26%). 7.3% did not read the written informed consent form at all. 27% of the patients considered the informed consent material too long. In survey 2, most patients took 15–30 min (41%) or less than 15 min (38%). 11.5% of the respondents did not read the written informed consent form at all. In both cohorts, elderly patients estimated their required reading time quite long, whereas young patients reported a rather short reading time.

Regarding the time given to consider the proposed therapy, 55% of the patients were satisfied in survey 1 and 45% of the patients were satisfied in survey 2. Each 11% stated that the time given was not sufficient.

#### Comprehension and satisfaction

While patients in survey 1 were asked about the purpose of the trial they were treated in, patients in survey 2 were asked instead about the therapy goal. 89% of the patients in cohort 1 reported knowing the purpose of the trial, whereas 9% and 2%, respectively, were only partially or not at all aware of the purpose. In cohort 2, 75% of the patients stated that they knew the treatment goal, whereas this remained partially or completely unclear for 18% and 7%, respectively. The statement “chances and risks of the therapy were presented well” was affirmed in both cohorts by more than 70% of the patients. More than 80% of the patients were able to name their disease correctly in both cohorts. Patients from cohort 1 were asked during their interview if they could recall the exact numbers for common and uncommon side effects, which was correctly answered by one-third of the patients. Especially elderly patients and patients with a low school education frequently gave wrong answers here. Only 7% of the patients could explain the principle of a randomized trial correctly.

In both cohorts, each at least 70% of the patients considered the written informed consent form as decently comprehensible and well structured. While 16% of the patients from cohort 1 stated that the consent form contained difficult words, this was slightly lower in cohort 2, at 13%. Our precedent work demonstrated that patients with low school education had significantly more comprehension troubles than patients with at least 10 or 13 years of school education [11]. To analyze this for patients treated in clinical trials (cohort 1), we found no differences between the number of school years and comprehensibility of the information material, indicating that the informed consent material for clinical trials was decently comprehensible for all levels of school formation (*r* = 0.088, *p* = 0.440, correlation according to Spearman).

#### Patients’ attitude towards consent

**Fig. 1:**
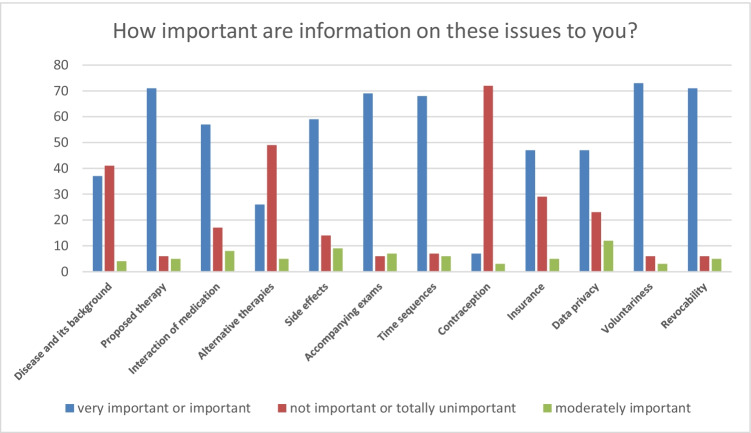
Distribution of subjective relevance of information for patients in cohort 1 (absolute numbers)

**Fig. 2:**
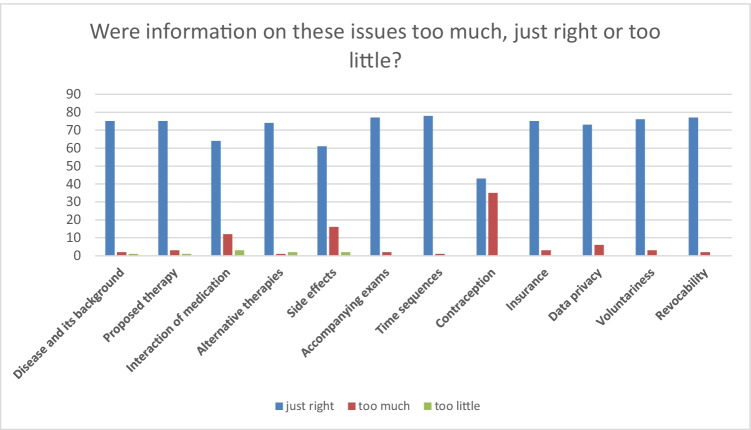
Distribution of sufficient display of information for patients in cohort 1 (absolute numbers)

**Fig. 3:**
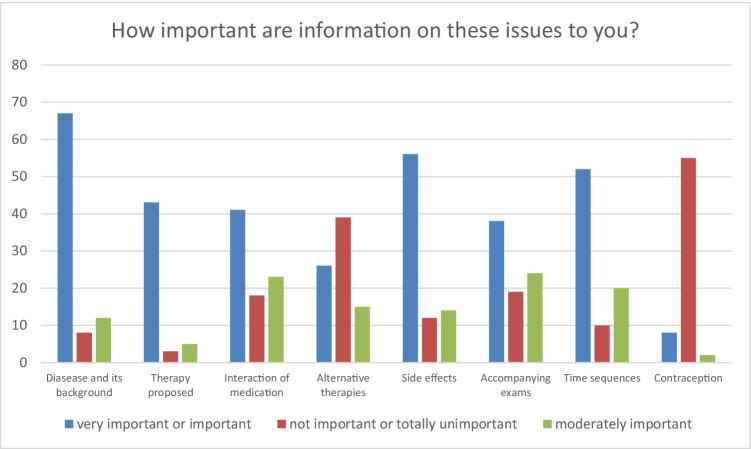
Distribution of subjective relevance of information for patients in cohort 2 (absolute numbers)

**Fig. 4:**
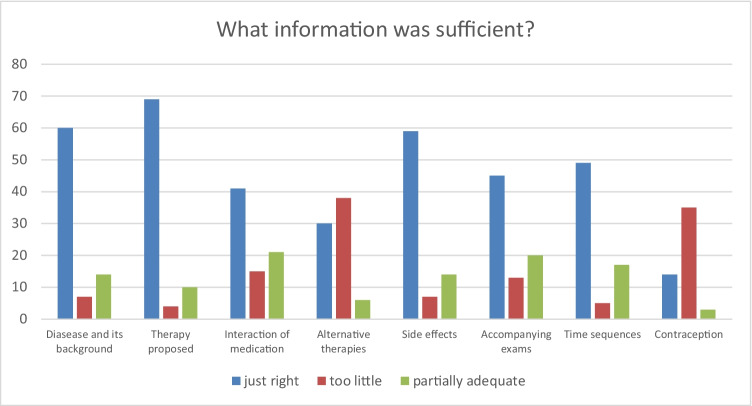
Distribution of sufficient display of information for patients in cohort 2 (absolute numbers)

Satisfaction with the provided information was equally very high. In both surveys, patients were asked about the most relevant information and if the information were sufficiently provided. Figures 1 and 2 show the distribution of subjective relevance of information and if the information were provided sufficiently for patients in survey 1; Figs. 3 and 4 show the same results for patients in survey 2.

Patients in survey 1 considered information about voluntariness concerning their trial participation and the possibility of revocability most relevant, followed by information on the proposed therapy, accompanying exams, and time sequences. Information about the disease and its background, alternative therapies, and contraception were considered less or not important. 44% of the patients stated that information about contraception was too extensive, most of them were male (63%). About 10% of the patients further stated that information about side effects and interaction of medication was too extensive. In total, 21% of the patients affirmed the statement “The consent form could have been shorter.”

Most relevant information for the patients in survey 2 were (in descending order) information about the disease and its background, side effects, time sequences, and the therapy proposed. Information about contraception, alternative therapies, accompanying exams, and interaction of medication were considered less important. The follow-up evaluation with 52 patients showed general satisfaction with the information provided. However, 25% of the patients lacked information on some parts, 16% lacked information in general and noticeably, and only 40% of the patients were satisfied with information on alternative therapies [16].

Patients’ perception of the informed consent process differed quite much in both cohorts. The statement “I just wanted to sign the informed consent without further information” was affirmed by more than twice as many patients in survey 2 (29% versus 11% in survey 1). In survey 2, patients who stated that they just wanted to sign the informed consent did not read the form completely either (survey 2: *r* =  − 0.376, *p* < 0.001, correlation according to Spearman). This was not applicable for patients from survey 1 (*r* =  − 0.032, *p* = 0.790)). The probability of receiving many additional information from nurses was about one-third higher for patients in survey 1 than for patients from survey 2 (absolute risk difference of + 29.7% (CI: + 15.5%; + 42.8%)).

## Discussion

In this study, we compared patients’ comprehension and satisfaction of current informed consent procedures in routine clinical care with the level of comprehension and satisfaction of patients treated within clinical trials at the same comprehensive cancer center in a real-world scenario.

To evaluate the effectiveness of an informed consent process, individual comprehension must be divided into subjective and objective, testable comprehension [17]. Most patients in both surveys indicated a high level of comprehension, which, considered from an objective point of view, was not always accurate. For example, 20% of the patients in both cohorts were not able to name their cancer diagnosis correctly. Additionally, uncertainty about the proposed treatment was present in both cohorts. About 11% of the patients treated within a clinical trial knew the purpose of the trial only partially or not at all, indicating a lack of comprehension. However, it must be pointed out that during the interview patients could rate the information given (too much, just right, too little) and on average, all participants rated the information given as “just right.” Patients only rated the topic “contraception” as too extensive, which might be an irrelevant issue for many patients since the median age at study inclusion was 58 years. Overall, results might be too positive here due to an interviewer bias. Additionally, social desirability—both during filling in the questionnaire and during the interview in cohort 1—might have also influenced the results. Even though the interviewer reassured each patient about the confidentiality of the answers, the procedure of pseudonymized analysis, and guarantee of data preservation, social desirability cannot be ruled out here. The partial uncertainty could also be linked to the fact that most patients in survey 1 took very little time reading the informed consent form: More than 70% of the patients reported they read it completely. 40% indicated they read it in less than 15 min, yet the material contained up to 40 pages. Objectively, patients must have rather quickly skimmed the material than really reading it carefully in that short amount of time. In contrast to this, the level of uncertainty was higher in patients from cohort 2: for 18% of the patients, the treatment goal remained partially unclear and 7% of the patients did not know the treatment goal at all. Subgroup analysis showed that these patients received palliative treatment, which implies communication weaknesses between the treating oncologists and the patients, since full disclosure of a life-threatening diagnosis can be challenging and misconception in cancer communication is a common problem [18]. Retrospectively, 40% of the patients missed information about alternative therapies. About 20% of the patients from both surveys had troubles understanding the consent form in some parts. However, the statement “I had to ask a lot of questions to understand the informed consent form” was affirmed by only 10% in each cohort, indicating a potential disinterest to understand each detail. This might hold true especially for patients in survey 2, since almost three times as many patients receiving standard cancer therapy affirmed the statement “I just wanted to sign the informed consent form” (29% in survey 2 versus 11% in survey 1), indicating that participants in clinical trials prefer to know more details than patients receiving standard care. However, each 10% of the patients in both cohorts did not read the information material at all. Noticeably, patients’ perception of the informed consent talk differed quite much: in both cohorts, patients were asked whether they felt reassured after their informed consent talk. Whereas 48.5% of the patients from survey 2 fully agreed here, only 12% of the patients from survey 1 agreed. This might be additional evidence for uncertainty or comprehension troubles.

Jefford et al. performed a similar study to examine knowledge and satisfaction regarding the informed consent process concerning cancer clinical trials with 102 patients being treated in 27 different therapeutic trials [19]. In line with our findings, the authors stated that knowledge regarding cancer clinical trials was generally good, but significant information was frequently missing; e.g., each 12% of the trial participants missed information on the specific cancer being studied, the reason for research, or other treatment options available [19]. Importantly, satisfaction correlated with perceived but not objective understanding [19], what we could also demonstrate in our surveys.

Various attempts to overcome comprehension limitations have already been discussed. A larger font size and stronger contrasts as well as a concise and simpler language may help to improve the readability [20, 21]. Multimodal approaches to optimize the quality of the informed consent process—not only in the setting of cancer therapy—have shown promising results, e.g., the use of touch-screen tablets, animated videos, slideshows with voice-over or comics [22, 23], or feedback interventions [20]. However, none of these attempts has yet been implemented into the real-world scenario of patient education, even though we have been aware of these shortcomings for years.

## Conclusions

In summary, we can state that regardless of a standard of care cancer treatment or a treatment within a clinical cancer trial, patients’ self-assessment of comprehension after informed consent often differs from the actual objective comprehension. However, satisfaction with the information provided was very high in both cohorts. Our data support the hypothesis that extensive informed consent procedures for clinical cancer trials are not associated with a higher level of satisfaction or objectifiable comprehension and therefore, the benefit for patients is limited. Unmet needs in terms of individual capacities and expectations need to be better addressed. In a future project, we aim to design patient-centered, modular information and consenting material, which can be used and adapted to patients’ individual needs and capacities, considering different age and education levels.

### Study limitations

Our study has some limitations that have to be acknowledged. First, the questionnaire used was developed, piloted, and validated at our own hospital, but has not been validated nationally or internationally. The questionnaire has been modified for a more user-friendly design and some redundant questions have been removed for the second survey. However, the principal questions on comprehension and satisfaction remained the same and we therefore believe that its feasibility is sufficiently established. Second, only patients in survey 1 received a structured interview and only patients in survey 2 received a follow-up questionnaire, which is a methodical weakness. The reason for this modification was that the additional benefit of the interview was limited since many of the questions were identical or similar to those from the questionnaire—and so were the answers. There may be some room for an interviewer bias, since patients may have chosen more positive answers due to social desirability. Therefore, we decided to perform a follow-up questionnaire instead to generate new answers and to have more trustable insight of patients’ satisfaction and comprehension retrospectively. There may be selection bias as well, since patients in survey 1 all had a curative therapy goal whereas patients in survey 2 also had adjuvant and palliative therapy goals. Moreover, patients in survey 1 received more extensive educational material than patients from survey 2, who received the standardized material, which may have led to a higher number of patients feeling not reassured after the informed consent talk in survey 1. Last, our findings may only hold true for German patients and may not be generalized for patients from other countries.
